# Active Versus Expectant Management for Preterm Premature Rupture of Membranes at 34–36 Weeks of Gestation and the Associated Adverse Perinatal Outcomes

**DOI:** 10.1055/s-0040-1718954

**Published:** 2020-11-30

**Authors:** Malú Flôres Ferraz, Thaísa De Souza Lima, Sarah Moura Cintra, Edward Araujo Júnior, Caetano Galvão Petrini, Mario Sergio Silva Gomes Caetano, Marina Carvalho Paschoini, Alberto Borges Peixoto

**Affiliations:** 1 Department of Obstetrics and Gynecology, Universidade Federal do Triângulo Mineiro, Uberaba, MG, Brazil; 2 Service of Gynecology and Obstetrics, Hospital Universitário Mário Palmério, Universidade de Uberaba, Uberaba, MG, Brazil; 3 Department of Obstetrics, Escola Paulista de Medicina, Universidade Federal de São Paulo, São Paulo, SP, Brazil; 4 Medical course, Universidade Municipal de São Caetano do Sul, São Paulo, SP, Brazil

**Keywords:** preterm premature rupture of membranes, antibiotic prophylaxis, maternal morbidity, neonatal morbidity, ruptura prematura de membranas, profilaxia antibiótica, morbidade materna, morbidade neonatal

## Abstract

**Objective** To compare the type of management (active versus expectant) for preterm premature rupture of membranes (PPROM) between 34 and 36 + 6 weeks of gestation and the associated adverse perinatal outcomes in 2 tertiary hospitals in the southeast of Brazil.

**Methods** In the present retrospective cohort study, data were obtained by reviewing the medical records of patients admitted to two tertiary centers with different protocols for PPROM management. The participants were divided into two groups based on PPROM management: group I (active) and group II (expectant). For statistical analysis, the Student *t* -test, the chi-squared test, and binary logistic regression were used.

**Results** Of the 118 participants included, 78 underwent active (group I) and 40 expectant management (group II). Compared with group II, group I had significantly lower mean amniotic fluid index (5.5 versus 11.3 cm, *p* = 0.002), polymerase chain reaction at admission (1.5 versus 5.2 mg/dl, *p* = 0.002), time of prophylactic antibiotics (5.4 versus 18.4 hours, *p* < 0.001), latency time (20.9 versus 33.6 hours, *p* = 0.001), and gestational age at delivery (36.5 versus 37.2 weeks, *p* = 0.025). There were no significant associations between the groups and the presence of adverse perinatal outcomes. Gestational age at diagnosis was the only significant predictor of adverse composite outcome (x ^2^ [1] = 3.1, *p* = 0.0001, R ^2^ Nagelkerke = 0.138).

**Conclusion** There was no association between active versus expectant management in pregnant women with PPROM between 34 and 36 + 6 weeks of gestation and adverse perinatal outcomes.

## Resumo

**Objetivo** Comparar o tipo de manejo (ativo versus expectante) para ruptura prematura de membranas (PPROM, na sigla em inglês) entre 34 e 36 semanas e 6 dias de gestação e os resultados perinatais adversos relacionados, em 2 hospitais terciários do sudeste brasileiro.

**Métodos** No presente estudo de coorte retrospectivo, os dados foram obtidos através da revisão dos prontuários de gestantes internadas em dois centros terciários com protocolos diferentes para o seguimento da PPROM. As gestantes foram divididas em dois grupos com base no manejo da PPROM: grupo I (ativo) e grupo II (expectante). Para análise estatística, foram utilizados o teste t de Student, qui-quadrado e regressão logística binária.

**Resultados** Das 118 gestantes incluídas, 78 foram submetidas a tratamento ativo (grupo I) e 40 a seguimento expectante (grupo II). Comparado ao grupo II, o grupo I apresentou índice de líquido amniótico médio significativamente menor (5,5 versus 11,3 cm, *p* = 0,002), reação em cadeia da polimerase na admissão (1,5 versus 5,2 mg/dl, *p* = 0,002), tempo de antibióticos profiláticos (5,4 versus 18,4 horas, *p* < 0,001), tempo de latência (20,9 versus 33,6 horas, *p* = 0,001) e idade gestacional no parto (36,5 versus 37,2 semanas, *p* = 0,025). Não houve associações significativas entre os grupos e a presença de resultados perinatais adversos. A idade gestacional no diagnóstico foi o único preditor significativo de desfecho composto adverso (x ^2^ [1] = 3,1, *p* = 0,0001, R ^2^ Nagelkerke = 0,138).

**Conclusão** Não houve associação entre manejo ativo e expectante em gestantes com PPROM entre 34 e 36 semanas e 6 dias de gestação e resultados perinatais adversos.

## Introduction

The term premature amniorrexis or premature rupture of membranes (PROM) is defined as the spontaneous rupture of membranes before the initiation of labor. It can occur in pregnancies prior to term between 20 and 37 weeks of gestation, referred to as preterm PROM (PPROM), or in term pregnancies. 1 2 Premature rupture of membranes occurs in ∼ 10% of all births, in ∼ 7% of term and in 3% of preterm pregnancies. Cases of PROM occur in 0.5% of preterm births before 27 weeks; in 1.5% between 27 and 34 weeks; and in 1% between 34 and 37 weeks. 3

Premature rupture of membranes in term pregnancies presents with complications in ∼ 8% of the cases. It is usually followed by rapid spontaneous labor within 24 hours, 4 with 79% of cases in 12 hours and 95% in 24 hours. 5 6 In term pregnancies, active management with immediate delivery is associated with a lower incidence of adverse perinatal outcomes compared with expectant management. 7 On the other hand, the ideal management of patients with PPROM before 37 weeks of gestation is not well defined. 8

The management of PPROM between 34 and 37 weeks of gestation is influenced by several factors such as gestational age, presence of infection (maternal or fetal sepsis), time of amniorrexis, and availability of intensive neonatal care. An accurate determination of gestational age and the maternal, fetal, and neonatal risks must be considered in the management of these pregnant women. 9 There is great international variation in the management of pregnancies complicated by PPROM close to term (between 34 and 37 weeks). The American College of Obstetricians and Gynecologists and the Royal College of Obstetricians and Gynecologists recommend that after 34 weeks, all cases should be actively managed and delivery should be considered. 10 11 We acknowledge these recommendations, yet they are based on limited scientific evidence. 10 12

In the management of PPROM, the risk of postponing delivery must be balanced against the risk of iatrogenic prematurity. In pregnancies earlier than 30 weeks, in the absence of signs of infection, expectant management is preferred due to the high risk of complications associated with extreme prematurity. 12 To reduce the risk of maternal and fetal infections, the use of prophylactic antibiotics in cases of PPROM before 34 weeks is recommended. 13 The use of prophylactic antibiotics in PPROM is associated with an increased gestational age and reduced maternal and neonatal infection rates. 13 14 15 However, there is little evidence regarding antibiotic prophylaxis in near-term PPROM and term PROM.

In developing countries, the lack of neonatal intensive care unit (NICU) access is a limiting factor in the proper management of PPROM. For this reason, as well as the lack of robust evidence regarding the best management of near-term PPROM, clinical practices vary greatly between tertiary institutions.

The objective of the present study was to compare the type of management (active or expectant) for near-term PPROM (between 34 and 36 + 6 weeks of gestation) and adverse perinatal and composite outcomes in 2 tertiary hospitals in the southeast of Brazil.

## Methods

The present retrospective cohort study was performed by reviewing the medical records of pregnant women admitted to the Hospital das Clínicas of the Universidade Federal do Triângulo Mineiro (UFTM, in the Portuguese acronym) and to the Hospital Universitário Mário Palmério of the Universidade de Uberaba (UNIUBE, in the Portuguese acronym) in Uberaba, state of Minas Gerais, southeast Brazil, from January 2014 to January 2018. The patients included in the present study were divided into 2 groups: group I included active management and group II expectant management. The study was approved by the Ethics Committee of the UFTM (CAAE: 92166618.1.0000.5154), and patient consent was not necessary since it was a retrospective study.

The present study included all patients with singleton pregnancies with spontaneous and confirmed PPROM (by clinical, laboratory, and/or imaging tests) between 34 and 36 + 6 weeks. The gestational age of each patient was based on her last menstrual period (LMP) and confirmed by obstetric ultrasonography in the 1 ^st^ trimester.

Twin pregnancies and those associated with maternal chronic diseases such as chronic arterial hypertension, pre-eclampsia, systemic lupus erythematosus, diabetes mellitus, and thrombophilia were excluded. Pregnancies with fetal malformations detected at the 1 ^st^ or 2 ^nd^ trimester scan and chromosomal abnormalities confirmed by fetal karyotype were also excluded.

The UFTM protocol dictates that pregnant women with PPROM between 34 and 36 + 6 weeks of gestation are actively managed (group I). After receiving a confirmed diagnosis of PPROM, the patient is admitted to the hospital and the doctor orders a blood count, polymerase chain reaction (PCR), urine type 1, and urine culture, as local protocol. Fetal monitoring is performed by cardiotocography and obstetric ultrasound with Doppler. The use of antibiotic prophylaxis is recommended after 6 hours of PPROM, specifically crystalline penicillin administered as a 5 million IU loading dose and 5 million IU at 4/4 hours maintenance dose until delivery. Once the pregnant women were admitted to the obstetrics unit, the induction of labor or delivery was immediately indicated. The mode of delivery is chosen according to obstetric indication.

According to the UNIUBE protocol, pregnant women diagnosed with PPROM between 34 and 36 + 6 weeks of gestation are hospitalized and monitored expectantly (group II). Maternal monitoring occurs through clinical evaluation daily and laboratory every 3 days. For maternal infectious screening, the following tests are ordered: blood count, PCR, urine type 1, urine culture, culture for β-hemolytic streptococci (rectal and vaginal swabs), and bacterioscopy of vaginal secretions. Fetal monitoring occurs through daily cardiotocography and obstetric ultrasound with Doppler. Antibiotic prophylaxis is always performed upon admission immediately after the diagnosis of PPROM. In the absence of maternal hypersensitivity, crystalline penicillin is recommended with a 5 million IU loading dose and 2.5 million 4/4 hours maintenance dose for 7 days. Delivery is indicated according to the obstetric and pediatric conditions and the availability of a NICU, and is immediate when there are clinical or laboratory signs of maternal infection and chorioamnionitis.

In both centers, the diagnosis of PROM was made in the presence of the following clinical/laboratory signs: typical history of fluid loss by the external cervical os, clinical presence of a moist vulva, visualization of fluid in the vaginal sac during the specular examination, and a positive crystallization test. Premature rupture of membranes was also diagnosed through amniocentesis, specifically by observing the contrast (vitamin B12) from the vagina of the patient around between 30 and 60 minutes after its injection into the amniotic cavity. In both methods, obstetric ultrasound was not used for the diagnosis of PROM; however, in the presence of oligohydramnios associated with suggestive and/or doubtful clinical signs, patients were treated for PROM. Oligohydramnios was defined by an amniotic fluid index (AFI) < 5 cm or the largest pocket of amniotic fluid < 2 cm.

Neonatal infection was diagnosed by positive culture from a sample collected in a normally sterile location associated with clinical signs of infection or elevated neonatal PCR (> 10 mg/dl), or with a chest X-ray indicative of lung infection. Neonatal sepsis was diagnosed when culture of peripheral blood or cerebrospinal fluid was positive and associated with clinical symptoms of infection and PCR > 10 mg/dl. Clinical chorioamnionitis was defined as an axillary temperature > 38°C and no other cause of diagnosed fever, in addition to PCR >10 mg/dl or fetal tachycardia.

The following variables were evaluated: Apgar score at the 1 ^st^ and 5 ^th^ minutes, birthweight, length of stay in the NICU, presence of neonatal infection (neonatal sepsis), need for oxygen therapy within 24 hours of delivery, use of surfactant, number of deliveries between 48 hours and 7 days after the diagnosis of PPROM, presence of chorioamnionitis and maternal sepsis, time between PPROM diagnosis and labor (latency period), type of delivery, and demographic data of the pregnant women (age, ethnicity, parity, smoking, drinking, and the presence of comorbidities). Perinatal adverse outcomes included the following: chorioamnionitis, maternal sepsis, neonatal sepsis, Apgar score < 7 at the 5 ^th^ minute, admission to the NICU, use of surfactant, and oxygen therapy after delivery. Patients were categorized as having an adverse perinatal outcome based on the presence of at least one adverse perinatal outcome.

The data were analyzed in an Excel 2007 spreadsheet (Microsoft Corp., Redmond, WA, USA) using the SPSS 20.0 (SPSS Inc., Chicago, IL, USA) and Prism GraphPad 7.0 (GraphPad Software, San Diego, CA, USA). Quantitative variables were first subjected to the normality test (Kolmogorov-Smirmov). Continuous variables were presented as means with standard deviations (SDs), and their differences were assessed by the Student *t* -test. Categorical variables were described as absolute values and percentages, and were represented in tables. To study the differences between categorical variables and their proportions, the chi-squared test was used. Binary logistic regression was performed to determine the best predictors of adverse perinatal and composite outcomes in each of the groups. Using logistic regression analyses, odds ratios (ORs) were determined for the development of adverse perinatal and composite outcomes for the tested variables. The level of significance (p) for all tests was 0.05.

## Results

**Fig. 1:**
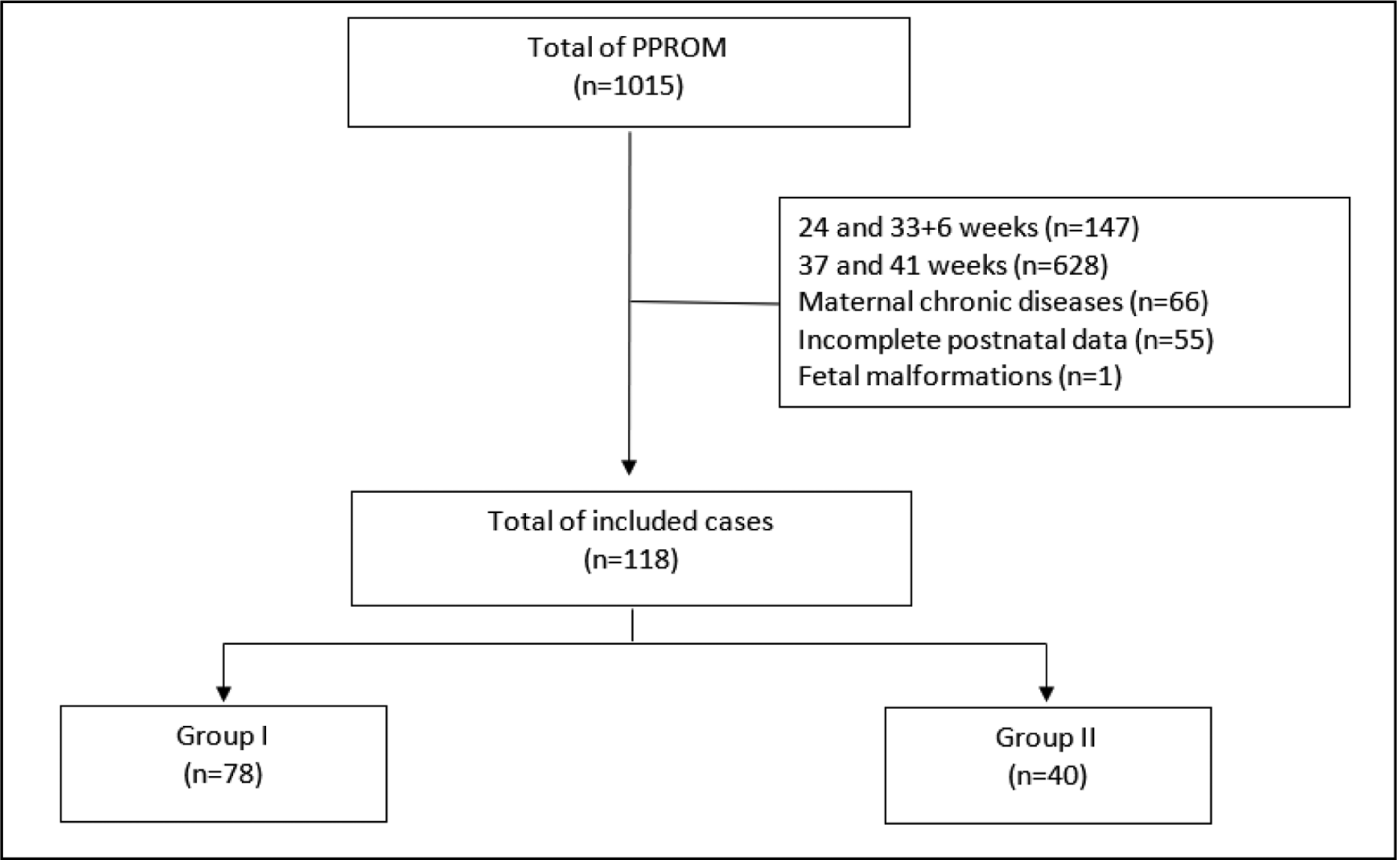
Flowchart of the cases included in the study.

From January 2014 to January 2018, 1,015 patients at 24 weeks gestation or later were diagnosed with PPROM in the 2 participating centers. Among the 1,015 patients, 14.5% (147/1015) were between 24 and 33 + 6 weeks of gestation, 23.6% (240/1015) between 34 and 36 + 6 weeks, and 61.9% (628/1015) between 37 and 41 weeks. Among the 240 patients between 34 and 36 + 6 weeks, 122 cases were excluded due to maternal chronic diseases ( *n* = 66), fetal malformations ( *n* = 1), and incomplete postnatal data ( *n* = 55). For the final statistical analysis, 118 participants were included, divided into group I ( *n* = 78) and group II ( *n* = 40) ( Fig. 1 ).

Table 1 shows the maternal demographic data and perinatal outcomes of the studied population. Significant differences were observed between the groups regarding PCR at admission ( *p* = 0.005), time of prophylactic antibiotics ( *p* < 0.001), latency time ( *p* = 0.001), and gestational age at delivery (p ≤ 0.001). Compared with group II, group I had significantly lower mean AFI (5.5 versus 11.3 cm, *p* = 0.002), PCR at admission (1.5 versus 5.2 mg/dl, *p* = 0.002), time of prophylactic antibiotics (5.4 versus 18.4 hours, *p* < 0.001), latency time (20.9 versus 33.6 hours, *p* = 0.001), and gestational age at delivery (36.5 versus 37.2 weeks, *p* = 0.025).

**Table 1:** Maternal demographic data and perinatal outcomes

	Group I ( *n* = 78)	Group II ( *n* = 40)	p-value
Maternal age (years old)	23.7 (5.5)	22.7 (6.0)	0.379 †
Ethnicity			0.366 §
White	42.59% (33/77)	43.6% (17/39)	
Mixed	49.4% (38/77)	51.3% (20/39)	
Black	7.8% (6/77)	2.6% (1/39)	
Asian	0% (0/77)	2.6% (1/39)	
Tobacco use			0.565 §
Yes	11.1% (8/72)	7.7% (3/39)	
No	88.9% (64/72)	92.3% (36/39)	
Alcohol use			0.202 §
Yes	4.2% (3/72)	0% (0/38)	
No	95.8% (69/72)	100% (38/38)	
Number of pregnancies	1.9 (1.2)	1.9 (1.0)	0.876 †
Parity	0.70 (1.2)	0.60 (0.9)	0.456 †
GA at admission (weeks)	35.6 (0.7)	35.8 (0.7)	0.097 †
AFI (cm)	5.50 (3.4)	11.30 (3.5)	0.002 †
PCR at admission (mg/dl)	1.50 (2.4)	5.2 (3.1)	0.002 †
Time of prophylactic antibiotics (hours)	5.40 (6.5)	18.4 (16.0)	<0.001 †
Latency time (hours)	20.9 (17.6)	33.60 (24.0)	0.001 †
Type of delivery			0.303 §
Vaginal	71.8% (56/78)	62.5% (25/40)	
Cesarean section	28.2% (22/78)	37.5% (15/40)	
Birthweight (grams)	2577.4 (392.6)	2650 (416.3)	0.354 †
GA at delivery (weeks)	36.50 (0.9)	37.20 (1.1)	<0.001 †
Apgar score at 1 ^st^ minute	8.30(1.3)	8.30 (1.3)	0.941 †
Apgar score at 5 ^th^ minute	8.9 (0.5)	9.0 (0.8)	0.941 †
Time of NICU (hr)	306 (231.3)	191.4 (239.9)	0.459 †

Abbreviations: AFI, amniotic fluid index; GA, gestational age; NICU, neonatal intensive care unit; PCR, polymerase chain reaction.

† Student *t* -test: mean (standard deviation).

§ Chi-squared percentage (absolute number), *p* < 0.05.

In group II, we calculated the Pearson correlation coefficient to assess whether there were correlations between the time of prophylactic antibiotics, gestational age at admission, and the latency time. We did not include patients from group I in this analysis because active management could influence the results.

**Fig. 2:**
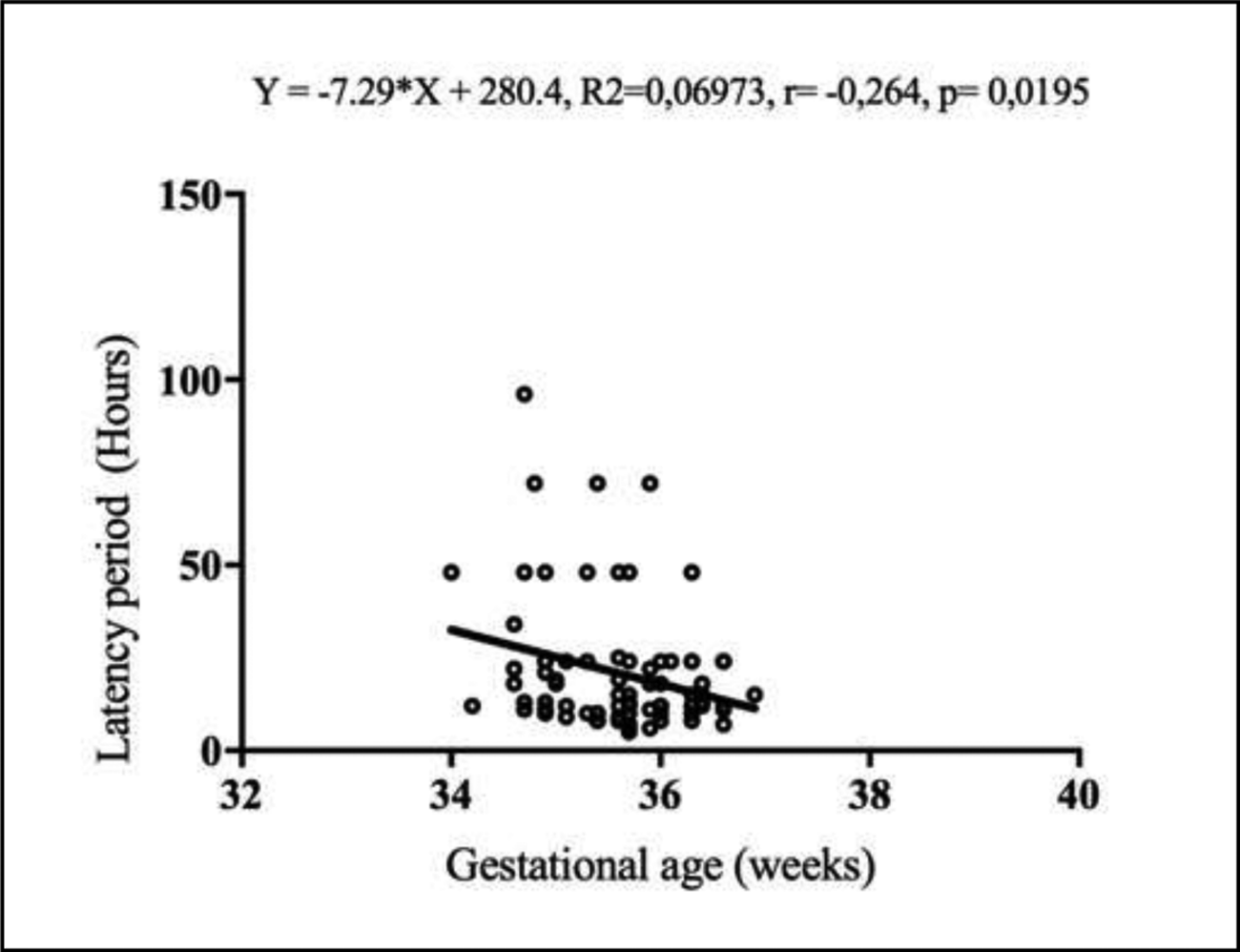
Scatter plot showing the relationship between gestational age at the time of diagnosis of preterm premature rupture of membranes (PPROM) in pregnant women monitored expectantly between 34 and 36 + 6 weeks and the latency period. Pearson correlation coefficient ( *p* < 0.05).

In pregnant women between 34 and 36 + 6 weeks monitored expectantly, we observed no significant correlation between the time of prophylactic antibiotics and the latency period (r = 0.215, *p* = 0.218), and a weak but significant negative correlation between gestational age at admission and the latency time (r = -0.264, *p* = 0.0195). In these patients, an increase of 1 week in gestational age at the time of diagnosis reduced the latency time by 7.29 hours ( Fig. 2 ).

There were no significant differences between the groups regarding the presence of adverse perinatal results ( Table 2 ).

**Table 2:** Adverse perinatal outcomes in patients with premature rupture of membranes between 34 and 36 + 6 weeks undergoing active (Group I) and expectant (Group II) management

	Group I ( *n* = 78)			Group II ( *n* = 40)			X ^2^	*p-value*
	n	N	%	n	N	%		
Apgar score at 5th min < 7							1.75	0.185
Yes	7	78	9.0	1	40	2.5		
No	71	78	91.0	39	40	97.5		
NICU							2.04	0.153
Yes	4	78	5.1	5	40	12.5		
No	74	78	94.9	35	40	87.5		
Needed oxygen within 24h							0.587	0.444
Yes	17	78	21.8	12	40	30.0		
No	61	78	78.2	28	40	70.0		
Surfactant								†
Yes	0	78	0.0	0	40	0.0		
No	78	78	100.0	40	40	100.0		
Fetal death								†
Yes	0	78	0.0	1	40	2.5		
No	78	78	100.0	39	40	97.5		
Neonatal death								†
Yes	0	78	0.0	0	40	0.0		
No	78	78	100.0	40	40	100.0		
Maternal death								†
Yes	0	78	0.0	0	40	0.0		
No	78	78	100.0	40	40	100.0		
Neonatal sepsis							0.479	0.489
Yes	2	78	2.6	2	40	5.0		
No	76	78	97.4	38	40	95.0		
Maternal sepsis								†
Yes	0	78	0.0	0	40	0.0		
No	78	78	100.0	40	40	100.0		
Chorioamnionitis							0.235	0.628
Yes	1	78	1.3	1	40	2.5		
No	77	78	98.7	39	40	97.5		
Composite neonatal outcome							0.668	0.414
Yes	18	78	23.1	12	40	30.0		
No	60	78	76.9	28	40	70.0		

Abbreviations: n, absolute number; N, total number assessed for each variable; NICU, neonatal intensive care unit.

Chi Square *p* < 0.005.

† It was not possible to perform statistical tests on variables with less than three patients in each category.

A binary logistic regression analysis was performed to identify the main predictors of adverse composite outcomes in patients with PPROM between 34 and 36 + 6 weeks of gestation, and gestational age at diagnosis was the only significant predictor (x ^2^ [1] = 3.1, *p* = 0.0001, R ^2^ Nagelkerke = 0.138). Although significant, gestational age at diagnosis explained only 13.8% of the adverse composite outcomes. Smoking ( *p* = 0.513), AFI ( *p* = 0.810), time of prophylactic antibiotics ( *p* = 0.360), and latency time ( *p* = 0.885) were not predictors of adverse composite outcomes. Table 3 contains the OR and the 95% confidence intervals (CIs) for each model tested.

**Table 3:** Predictors of adverse composite outcomes in patients diagnosed with premature rupture of membranes between 34 and 36 +6 weeks of gestation

	OR	95%CI	X ^2^	R ^2^ (Nagelkerke)	*p-value*
Tobacco use	1.6	0.33–8.19	0.43	0.006	0.513
GA at admission (weeks)	3.1	1.56–6.31	0.14	0.138	0.001
AFI (cm)	0.97	0.76–1.23	0.058	0.005	0.810
PCR at admission (mg/dl)	0.9	0.87–1.08	3.67	0.149	0.055
Time of prophylactic antibiotics (hours)	1.0	0.97–1.06	0.84	0.011	0.36
Latency time (hours)	1.0	0.98–1.02	0.02	0	0.885

Abbreviations: AFI, amniotic fluid index; CI, confidence interval; GA, gestational age; OR, odds ratio; PCR, polymerase chain reaction.

Binary logistic regression *p* < 0.05.

## Discussion

In evaluating two different protocols using active (group I) or expectant management (group II) for PPROM, we did not observe significant differences in adverse perinatal or composite outcomes between the groups. Gestational age at PPROM diagnosis was the only significant predictor of adverse composite outcomes.

We did not observe significant differences between the type of management and its association with the presence of an Apgar score < 7 at the 5 ^th^ minute, admission to the NICU, and oxygen use within 24 hours. This can be explained by the fact that the mean gestational age at delivery was later than 36 weeks of gestation for both groups. The need for oxygen at delivery is greater in newborns at 34 weeks than at 35 or 36 weeks. 16 Although not significant, group I had longer time of hospitalization in NICU compared with group II. We believe that the longer time in NICU was due to the different medical teams and protocols of both centers, since there was no significant difference in gestational age at the time of admission and adverse perinatal outcomes between the groups.

Although administered in different doses, antibiotic prophylaxis was used in the management of PPROM in both groups. In pregnancies earlier than 34 weeks of gestation, the use of antibiotics is one of the factors responsible for modifying the latency time. 17 In our study, we believe that the shorter duration of prophylactic antibiotics use in group I did not influence the earlier gestational age at delivery. We observed no significant correlation between the duration of prophylactic antibiotics and latency time period in patients monitored expectantly (group II). On the other hand, we observed a significant negative correlation between gestational age at diagnosis and the latency time. Thus, we believe that the earlier gestational age at delivery of the patients actively monitored (group I) was influenced by the type of management and the gestational age at hospitalization.

In patients with PPROM or PROM, the latency time between diagnosis and delivery is inversely proportional to gestational age at diagnosis. 7 9 18 Dagklis et al., 19 in a retrospective study performed between 24 and 36 + 6 weeks of gestation, demonstrated that a more advanced gestational age at the time of hospitalization was related to a shorter latency time. In this study, the median latency time was 5.2 hours but exceeded 48 hours in 65% of the cases. 18 Nayot et al., 20 in a prospective study performed between 25 and 36 weeks of gestation, observed extension of the latency time to > 72 hours in 67% of pregnancies between 25 and 28 weeks and in only 10% of pregnancies between 33 and 36 weeks. In our study, a one-week increase in gestational age at diagnosis represented a decrease of 7.29 hours in the latency time for patients monitored expectantly. A weak negative correlation (r = - 0.264) between the latency time and gestational age at diagnosis was likely due to the small number of cases included in this analysis. The mean difference of the latency time between groups was 37.8%. In our study, we did not analyze the cost of hospitalization of both strategies of management. However, the latency time was significantly higher in expectant management than in the active group. We believe that this strategy of management is not more expensive than active management, since there was no significant difference in the time of hospitalization in NICU and adverse perinatal outcomes. To correctly count the cost of expectant versus active management, it is necessary to carry out cost-effectiveness studies. In addition, we observed that gestational age at diagnosis was a significant predictor of adverse perinatal outcomes (OR: 3.1, 95%CI: 1.56–6.31, *p* = 0.001).

Regardless of the gestational age at diagnosis, intrauterine infection is the most relevant complication, and it is more frequent with earlier diagnosis and longer duration of PPROM. 13 The use of prophylactic antibiotics prolongs pregnancy and reduces maternal and neonatal morbidity. 13 14 15 However, there is little information about the use of prophylactic antibiotics in pregnancies with PROM at term or close to term. 13 Between 34 and 41 weeks, the use of prophylactic antibiotics in patients with PROM is used to prevent infection by β-hemolytic streptococci (GBS). 9 In developing countries, a swab for GBS is not always routine; thus, antibiotics should be administered in the presence of risk factors such as: gestational age earlier than 37 weeks, prolonged rupture of membranes (> 18 hours), an intrapartum temperature > 38°C, a previous child with GBS, and a urine culture positive for GBS. 21 To evaluate the effectiveness of prophylactic antibiotics in pregnancies with PROM at 36 weeks of gestation or later, Saccone et al. 22 performed a meta-analysis containing 5 trials with 2,699 total patients. Women who received prophylactic antibiotics had the same risk for chorioamnionitis (2.7% versus 3.7%; relative risk [RR]: 0.73), endometritis (0.4% versus 0.9%; RR: 0.44), maternal infection (3.1% versus 4.6%; RR: 0.48), and neonatal sepsis (1.0% versus 1.4%; RR: 0.69). In a systematic review including 1,801 PROM patients at 36 weeks of gestation or later, Wojcieszek et al. 23 also reported that there were no benefits of prophylactic antibiotics for pregnant women and newborns. Consistent with previous studies, 22 23 in PPROM patients between 34 and 36 + 6 weeks under different antibiotic prophylaxis and management strategies (active versus expectant), we did not observe differences between the groups and their incidences of neonatal sepsis, maternal sepsis, chorioamnionitis, and adverse perinatal outcomes. In addition, we observed that the duration of prophylactic antibiotics was not a significant predictor of adverse composite outcomes.

Although patients with PROM have a higher risk of perinatal complications than patients without rupture of membranes at the same gestational age, 18 24 25 the probability of the fetus being born alive with no morbidity later than 34 weeks of gestation is high. 26 This has encouraged active management in pregnancies past 34 weeks with some complications. However, in developing countries, the availability of NICUs is limited. Our study demonstrated that expectant management between 34 and 36 + 6 weeks of gestation with prophylactic antibiotics and maternal monitoring did not show adverse perinatal outcomes compared with active management after 34 weeks of gestation. Our results reinforce the possibility of expectant management between 34 and 36 weeks in the absence of ideal conditions for delivery.

Contrary to some previously published trials, 27 28 we did not exclude cases of labor induction due to conditions related to PPROM or cases with a lack of fetal maturity from the expectant management group. The small number of cases, the comparison of results in two different centers, and the retrospective nature of the analyses were the main limitations of our study. As this was a retrospective observational study, the power calculations of the applied tests was performed posteriori. The power to assess effects of the monitoring strategy on continuous variables, the associations between the monitoring strategy and adverse perinatal outcomes, and the ability to predict adverse composite outcomes was 90%, 83.3%, and 71.5%, respectively.

## Conclusion

In summary, in patients with PPROM between 34 and 36 + 6 weeks of gestation, there were no differences between the association of active or expectant management with adverse perinatal/composite outcomes.
